# (5*S*)-3-Chloro-5-[(1*R*,2*S*,5*R*)-2-isopropyl-5-methyl­cyclo­hex­yloxy]-4-(4-methyl­piperidin-1-yl)furan-2(5*H*)-one

**DOI:** 10.1107/S1600536811005216

**Published:** 2011-02-19

**Authors:** Xiao-Mei Wang, Jian-Hua Fu, Song-Liang Cai, Zhao-Yang Wang

**Affiliations:** a School of Chemistry and Environment, South China Normal University, Guangzhou 510006, People’s Republic of China

## Abstract

The title compound, C_20_H_32_ClNO_3_, was obtained *via* a tandem asymmetric Michael addition–elimination reaction of (5*S*)-3,4-dichloro-5-(l-menth­yloxy)furan-2(5*H*)-one and 4-methyl­piperidine in the presence of potassium fluoride. The furan­one ring is approximately planar [maximum atomic deviation = 0.022 (2) Å] while the cyclo­hexane ring adopts a chair conformation. Weak inter­molecular C—H⋯O hydrogen bonding is present in the crystal structure.

## Related literature

The title compound is a derivative of 4-amino-2(5*H*)-furan­one. For the biological activity of 4-amino-2(5*H*)-furan­ones, see: Lattmann *et al.* (2005 ▶); Prasad & Gandi (2010 ▶); Steenackers *et al.* (2010 ▶). For asymmetric Michael addition reactions of 2(5*H*)-furan­one and for the synthesis of the title compound, see: Song *et al.* (2009 ▶).
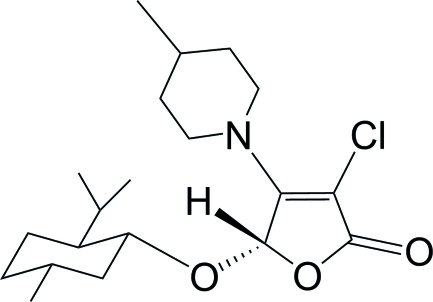


## Experimental

### 

#### Crystal data

C_20_H_32_ClNO_3_
*M* *_r_* = 369.92
Orthorhombic, 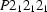

*a* = 9.187 (5) Å
*b* = 9.248 (5) Å
*c* = 24.987 (12) Å
*V* = 2122.9 (19) Å^3^
*Z* = 4
Mo *K*α radiation
μ = 0.20 mm^−1^
*T* = 298 K
0.32 × 0.30 × 0.28 mm

#### Data collection

Bruker APEXII area-detector diffractometer
Absorption correction: multi-scan (*SADABS*; Sheldrick, 1996 ▶) *T*_min_ = 0.940, *T*_max_ = 0.947
12264 measured reflections
4505 independent reflections
2620 reflections with *I* > 2σ(*I*)
*R*_int_ = 0.038

#### Refinement

*R*[*F* ^2^ > 2σ(*F* ^2^)] = 0.043
*wR*(*F* ^2^) = 0.112
*S* = 1.01
4505 reflections
231 parameters
24 restraints
H-atom parameters constrained
Δρ_max_ = 0.13 e Å^−3^
Δρ_min_ = −0.16 e Å^−3^
Absolute structure: Flack (1983 ▶), 1921 Friedel pairs
Flack parameter: 0.10 (8)

### 

Data collection: *APEX2* (Bruker, 2008 ▶); cell refinement: *SAINT* (Bruker, 2008 ▶); data reduction: *SAINT*; program(s) used to solve structure: *SHELXS97* (Sheldrick, 2008 ▶); program(s) used to refine structure: *SHELXL97* (Sheldrick, 2008 ▶); molecular graphics: *ORTEP-3 for Windows* (Farrugia, 1997 ▶); software used to prepare material for publication: *SHELXL97*.

## Supplementary Material

Crystal structure: contains datablocks global, I. DOI: 10.1107/S1600536811005216/go2003sup1.cif

Structure factors: contains datablocks I. DOI: 10.1107/S1600536811005216/go2003Isup2.hkl

Additional supplementary materials:  crystallographic information; 3D view; checkCIF report

## Figures and Tables

**Table 1:** Hydrogen-bond geometry (Å, °)

*D*—H⋯*A*	*D*—H	H⋯*A*	*D*⋯*A*	*D*—H⋯*A*
C4—H4⋯O2^i^	0.98	2.44	3.376 (3)	160
C18—H18*B*⋯O2^ii^	0.97	2.54	3.393 (4)	147

Symmetry codes: (i) 
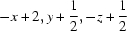
; (ii) 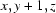.

**Fig. 1.**
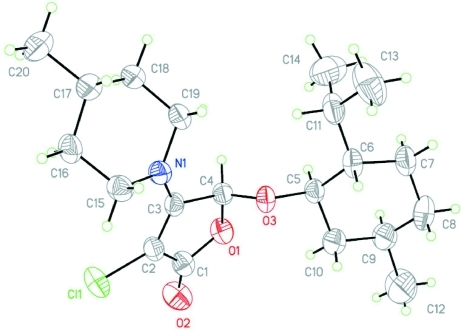
The molecular structure of the title compound showing the atom-labelling scheme. Ellipsoids are drawn at the 50% probability level.

**Fig. 2.**
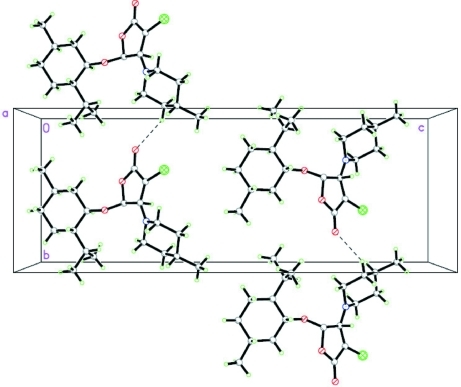
Perspective view of the crystal packing.

## supplementary crystallographic information

### Comment

2(5H)-furanones are heterocyclic carbonyl compounds, which are widespread in natural and in synthetic products (Prasad & Gandi, 2010; Steenackers *et al.*, 2010). 5-menthyloxy-3,4-dihalo-2(5H)-furanones, being a kind of chiral synthons, are widely used in asymmetric Michael addition-elimination tandem reactions (Song *et al.*, 2009). 4-amino-2(5H)-furanones show an antibiotic activity against Staphylococcus aureus (Lattmann *et al.*, 2005).

We are interested in the tandem Michael addition-elimination reaction of the chiral synthon 3,4-dichloro-5-(*S*)-(*l*-menthyloxy)-2(5H)-furanone and 4-methylpiperidine in the presence of potassium fluoride. The structure of the title compound (I) is illustrated in Fig. 1. The crystal structure of the title compound, which has four chiral centers (C4(*S*), C5(*R*), C6(*S*), C9(*R*)) contains a five-membered furanone ring and a six-membered cyclohexane ring connected each other *via* C4—O3—C5 ether bond. The furanone ring of C4—O1—C1—C2—C3 is approximately planar, whereas a six-membered cyclohexane ring displays a chair conformation. At the same time, the furanone ring is connected to piperidine heterocycle *via* C3—N1 bond.

### Experimental

The precursor 3,4-dichloro-5-(*S*)-(*l*-menthyloxy)-2(5H)-furanone was prepared according to the literature procedure (Song *et al.*, 2009). After the mixture of 3,4-dichloro-5-(*S*)-(*l*-menthyloxy)-2(5H)-furanone (2.0 mmol) and potassium fluoride (6.0 mmol) was dissolved in absolute tetrahydrofuran (2.0 mL) under nitrogen atmosphere, tetrahydrofuran solution of 4-methylpiperidine (2.0 mmol) was added. The reaction was carried out by stirring at room temperature for 24 h. Once the reaction was complete, the solvents were removed under reduced pressure. The residual solid was dissolved in dichloromethane. Then the combined organic layers from extraction were concentrated under reduced pressure, and the crude product was purified by silica gel column chromatography with the gradient mixture of petroleum ether and ethyl acetate to give the product yielding (I) 0.5685 g (76.8%).

### Refinement

H atoms were positioned in calculated positions with C—H = 0.93-0.98 Å and were refined using a riding model, with U_iso_(H) = 1.5U_eq_(C) for methyl and 1.2U_eq_(C) for the others.

### Crystal data

**Table:**

C_20_H_32_ClNO_3_	*F*(000) = 800
*M**_r_* = 369.92	*D*_x_ = 1.157 Mg m^−^^3^
Orthorhombic, *P*2_1_2_1_2_1_	Mo *K*α radiation, λ = 0.71073 Å
Hall symbol: P 2ac 2ab	Cell parameters from 2408 reflections
*a* = 9.187 (5) Å	θ = 2.7–19.8°
*b* = 9.248 (5) Å	µ = 0.20 mm^−^^1^
*c* = 24.987 (12) Å	*T* = 298 K
*V* = 2122.9 (19) Å^3^	Block, colourless
*Z* = 4	0.32 × 0.30 × 0.28 mm

### Data collection

**Table:**

Bruker APEXII area-detector diffractometer	4505 independent reflections
Radiation source: fine-focus sealed tube	2620 reflections with *I* > 2σ(*I*)
graphite	*R*_int_ = 0.038
φ and ω scans	θ_max_ = 26.8°, θ_min_ = 2.4°
Absorption correction: multi-scan (*SADABS*; Sheldrick, 1996)	*h* = −10→11
*T*_min_ = 0.940, *T*_max_ = 0.947	*k* = −11→10
12264 measured reflections	*l* = −31→31

### Refinement

**Table:**

Refinement on *F*^2^	Secondary atom site location: difference Fourier map
Least-squares matrix: full	Hydrogen site location: inferred from neighbouring sites
*R*[*F*^2^ > 2σ(*F*^2^)] = 0.043	H-atom parameters constrained
*wR*(*F*^2^) = 0.112	*w* = 1/[σ^2^(*F*_o_^2^) + (0.0492*P*)^2^ + 0.001*P*] where *P* = (*F*_o_^2^ + 2*F*_c_^2^)/3
*S* = 1.01	(Δ/σ)_max_ < 0.001
4505 reflections	Δρ_max_ = 0.13 e Å^−^^3^
231 parameters	Δρ_min_ = −0.16 e Å^−^^3^
24 restraints	Absolute structure: Flack (1983), 1921 Friedel pairs
Primary atom site location: structure-invariant direct methods	Flack parameter: 0.10 (8)

### Special details

**Table:**

Experimental. Data for (I): [α]^20°^_D_ = 96.2° (c 0.600, CH_3_CH_2_OH); ^1^H NMR (400 MHz, CDCl_3_, TMS): 0.769 (3H, *d*, *J* = 6.8 Hz, CH_3_), 0.831-0.934 (7H, *m*, CH, 2CH_3_), 0.981-1.166 (5H, *m*, CH_2_, CH_3_), 1.212-1.756 (9H, *m*, 3CH, 3CH_2_), 2.160-2.271 (2H, *m*, CH_2_), 2.974-3.090 (2H, *m*, CH_2_), 3.529-3.581 (1H, *m*, CH), 4.079-4.335 (2H, *m*, CH_2_), 5.762 (1H, *s*, CH), ESI-MS, *m*/*z* (%): Calcd for C_20_H_32_ClNO_3_^+^([M+H]^+^): 370.21(100.0), 372.20(32.0), Found: 370.29 (45.0), 372.33(15.0).
Geometry. All esds (except the esd in the dihedral angle between two l.s. planes) are estimated using the full covariance matrix. The cell esds are taken into account individually in the estimation of esds in distances, angles and torsion angles; correlations between esds in cell parameters are only used when they are defined by crystal symmetry. An approximate (isotropic) treatment of cell esds is used for estimating esds involving l.s. planes.
Refinement. Refinement of F^2^ against ALL reflections. The weighted R-factor wR and goodness of fit S are based on F^2^, conventional R-factors R are based on F, with F set to zero for negative F^2^. The threshold expression of F^2^ > 2sigma(F^2^) is used only for calculating R-factors(gt) etc. and is not relevant to the choice of reflections for refinement. R-factors based on F^2^ are statistically about twice as large as those based on F, and R- factors based on ALL data will be even larger.

### Fractional atomic coordinates and isotropic or equivalent isotropic displacement parameters (Å^2^)

**Table:**

	*x*	*y*	*z*	*U*_iso_*/*U*_eq_	
Cl1	0.60861 (9)	−0.13528 (9)	0.17373 (3)	0.0923 (3)	
C3	0.7320 (3)	0.0841 (3)	0.23600 (8)	0.0557 (6)	
C4	0.8425 (3)	0.0853 (3)	0.28119 (8)	0.0578 (6)	
H4	0.9252	0.1479	0.2725	0.069*	
C2	0.7123 (3)	−0.0578 (3)	0.22267 (9)	0.0621 (7)	
C1	0.8084 (3)	−0.1478 (4)	0.25291 (10)	0.0681 (7)	
C5	0.8604 (3)	0.1414 (3)	0.37500 (8)	0.0621 (6)	
H5	0.9630	0.1522	0.3650	0.074*	
C6	0.8125 (3)	0.2733 (3)	0.40633 (9)	0.0702 (8)	
H6	0.7085	0.2604	0.4136	0.084*	
C7	0.8883 (4)	0.2751 (4)	0.46068 (10)	0.0956 (9)	
H7A	0.9919	0.2897	0.4554	0.115*	
H7B	0.8517	0.3558	0.4815	0.115*	
C10	0.8414 (4)	0.0021 (4)	0.40651 (11)	0.0960 (11)	
H10A	0.8791	−0.0780	0.3856	0.115*	
H10B	0.7385	−0.0149	0.4124	0.115*	
C11	0.8269 (4)	0.4160 (4)	0.37596 (11)	0.0878 (10)	
H11	0.7799	0.4016	0.3412	0.105*	
C9	0.9194 (5)	0.0065 (4)	0.46042 (11)	0.1115 (13)	
H9	1.0238	0.0194	0.4538	0.134*	
C8	0.8648 (6)	0.1363 (5)	0.49177 (11)	0.1279 (14)	
H8A	0.7619	0.1245	0.4993	0.153*	
H8B	0.9160	0.1419	0.5257	0.153*	
C13	0.7463 (5)	0.5387 (4)	0.40314 (16)	0.1393 (16)	
H13A	0.7508	0.6236	0.3811	0.209*	
H13B	0.7906	0.5586	0.4371	0.209*	
H13C	0.6465	0.5117	0.4084	0.209*	
C14	0.9795 (6)	0.4590 (5)	0.36473 (16)	0.1399 (16)	
H14A	1.0262	0.4871	0.3975	0.210*	
H14B	0.9802	0.5388	0.3402	0.210*	
H14C	1.0308	0.3788	0.3493	0.210*	
C18	0.7583 (3)	0.4266 (3)	0.17613 (10)	0.0709 (7)	
H18A	0.8318	0.3755	0.1557	0.085*	
H18B	0.7946	0.5232	0.1833	0.085*	
C19	0.7345 (3)	0.3499 (3)	0.22804 (9)	0.0711 (7)	
H19A	0.8266	0.3401	0.2467	0.085*	
H19B	0.6696	0.4065	0.2504	0.085*	
C16	0.5572 (3)	0.2862 (3)	0.13592 (10)	0.0750 (8)	
H16A	0.4646	0.2930	0.1174	0.090*	
H16B	0.6226	0.2290	0.1140	0.090*	
C15	0.5354 (3)	0.2122 (3)	0.18891 (9)	0.0691 (7)	
H15A	0.4628	0.2643	0.2095	0.083*	
H15B	0.4996	0.1149	0.1830	0.083*	
C17	0.6204 (3)	0.4374 (3)	0.14313 (10)	0.0755 (8)	
H17	0.5498	0.4957	0.1631	0.091*	
C20	0.6467 (4)	0.5099 (4)	0.08935 (13)	0.1153 (13)	
H20A	0.5553	0.5251	0.0716	0.173*	
H20B	0.7073	0.4490	0.0676	0.173*	
H20C	0.6941	0.6012	0.0948	0.173*	
N1	0.6712 (2)	0.2061 (3)	0.21914 (8)	0.0635 (6)	
O3	0.77190 (15)	0.13329 (19)	0.32666 (5)	0.0606 (4)	
O1	0.88796 (18)	−0.0624 (2)	0.28665 (6)	0.0692 (5)	
O2	0.8292 (2)	−0.2765 (3)	0.25154 (9)	0.0958 (7)	
C12	0.8980 (9)	−0.1363 (5)	0.49012 (16)	0.193 (2)	
H12A	0.7961	−0.1514	0.4966	0.290*	
H12B	0.9489	−0.1329	0.5237	0.290*	
H12C	0.9355	−0.2142	0.4688	0.290*	

### Atomic displacement parameters (Å^2^)

**Table:**

	*U*^11^	*U*^22^	*U*^33^	*U*^12^	*U*^13^	*U*^23^
Cl1	0.0966 (5)	0.0925 (6)	0.0878 (5)	−0.0040 (5)	−0.0238 (4)	−0.0303 (4)
C3	0.0532 (14)	0.0716 (19)	0.0423 (11)	−0.0039 (13)	0.0037 (10)	−0.0047 (12)
C4	0.0566 (14)	0.0663 (19)	0.0507 (13)	−0.0053 (12)	−0.0014 (11)	−0.0038 (12)
C2	0.0612 (16)	0.072 (2)	0.0534 (13)	−0.0043 (14)	−0.0029 (12)	−0.0094 (13)
C1	0.0596 (15)	0.077 (2)	0.0677 (16)	0.0055 (16)	0.0053 (13)	−0.0158 (17)
C5	0.0649 (16)	0.0735 (17)	0.0478 (12)	−0.0011 (14)	−0.0084 (11)	−0.0026 (13)
C6	0.0673 (16)	0.092 (2)	0.0511 (13)	0.0086 (15)	−0.0004 (12)	−0.0171 (15)
C7	0.132 (3)	0.100 (2)	0.0542 (15)	−0.003 (2)	−0.0164 (17)	−0.0124 (17)
C10	0.132 (3)	0.092 (2)	0.0633 (16)	−0.020 (2)	−0.0195 (18)	0.0117 (17)
C11	0.126 (3)	0.078 (2)	0.0596 (16)	0.022 (2)	−0.0152 (17)	−0.0160 (16)
C9	0.173 (3)	0.094 (3)	0.0678 (18)	−0.024 (3)	−0.037 (2)	0.0227 (19)
C8	0.183 (4)	0.150 (3)	0.0514 (16)	−0.022 (3)	−0.016 (2)	0.002 (2)
C13	0.161 (4)	0.120 (3)	0.137 (3)	0.055 (3)	−0.042 (3)	−0.052 (3)
C14	0.165 (4)	0.103 (3)	0.152 (3)	0.002 (3)	0.053 (3)	0.013 (3)
C18	0.0791 (18)	0.0660 (18)	0.0678 (15)	−0.0153 (14)	−0.0104 (14)	0.0016 (14)
C19	0.0835 (19)	0.0686 (19)	0.0610 (15)	−0.0105 (16)	−0.0154 (14)	−0.0090 (14)
C16	0.0752 (18)	0.084 (2)	0.0663 (16)	−0.0017 (16)	−0.0243 (13)	−0.0002 (16)
C15	0.0592 (15)	0.0720 (18)	0.0760 (17)	−0.0043 (13)	−0.0121 (13)	−0.0002 (15)
C17	0.087 (2)	0.0695 (19)	0.0700 (15)	−0.0068 (17)	−0.0108 (15)	0.0073 (15)
C20	0.144 (3)	0.112 (3)	0.091 (2)	−0.029 (2)	−0.022 (2)	0.040 (2)
N1	0.0670 (13)	0.0634 (14)	0.0601 (11)	−0.0079 (12)	−0.0147 (10)	0.0039 (11)
O3	0.0557 (9)	0.0823 (12)	0.0436 (8)	0.0034 (9)	−0.0045 (7)	−0.0074 (9)
O1	0.0577 (10)	0.0779 (13)	0.0719 (10)	0.0108 (10)	−0.0093 (9)	−0.0086 (10)
O2	0.0908 (14)	0.0731 (15)	0.1237 (17)	0.0195 (12)	−0.0096 (12)	−0.0176 (14)
C12	0.339 (7)	0.136 (4)	0.105 (3)	−0.040 (5)	−0.072 (4)	0.049 (3)

### Geometric parameters (Å, °)

**Table:**

Cl1—C2	1.708 (2)	C8—H8B	0.9700
C3—N1	1.327 (3)	C13—H13A	0.9600
C3—C2	1.366 (4)	C13—H13B	0.9600
C3—C4	1.519 (3)	C13—H13C	0.9600
C4—O3	1.381 (3)	C14—H14A	0.9600
C4—O1	1.435 (3)	C14—H14B	0.9600
C4—H4	0.9800	C14—H14C	0.9600
C2—C1	1.429 (4)	C18—C19	1.495 (4)
C1—O2	1.206 (4)	C18—C17	1.514 (4)
C1—O1	1.367 (3)	C18—H18A	0.9700
C5—O3	1.458 (3)	C18—H18B	0.9700
C5—C6	1.514 (4)	C19—N1	1.468 (3)
C5—C10	1.520 (4)	C19—H19A	0.9700
C5—H5	0.9800	C19—H19B	0.9700
C6—C7	1.526 (4)	C16—C15	1.504 (3)
C6—C11	1.529 (4)	C16—C17	1.525 (4)
C6—H6	0.9800	C16—H16A	0.9700
C7—C8	1.516 (4)	C16—H16B	0.9700
C7—H7A	0.9700	C15—N1	1.460 (3)
C7—H7B	0.9700	C15—H15A	0.9700
C10—C9	1.526 (4)	C15—H15B	0.9700
C10—H10A	0.9700	C17—C20	1.521 (4)
C10—H10B	0.9700	C17—H17	0.9800
C11—C14	1.484 (5)	C20—H20A	0.9600
C11—C13	1.516 (5)	C20—H20B	0.9600
C11—H11	0.9800	C20—H20C	0.9600
C9—C8	1.519 (5)	C12—H12A	0.9600
C9—C12	1.527 (5)	C12—H12B	0.9600
C9—H9	0.9800	C12—H12C	0.9600
C8—H8A	0.9700		
			
N1—C3—C2	133.1 (2)	C11—C13—H13B	109.5
N1—C3—C4	120.7 (2)	H13A—C13—H13B	109.5
C2—C3—C4	106.1 (2)	C11—C13—H13C	109.5
O3—C4—O1	111.37 (19)	H13A—C13—H13C	109.5
O3—C4—C3	107.47 (18)	H13B—C13—H13C	109.5
O1—C4—C3	105.0 (2)	C11—C14—H14A	109.5
O3—C4—H4	110.9	C11—C14—H14B	109.5
O1—C4—H4	110.9	H14A—C14—H14B	109.5
C3—C4—H4	110.9	C11—C14—H14C	109.5
C3—C2—C1	110.4 (2)	H14A—C14—H14C	109.5
C3—C2—Cl1	130.7 (2)	H14B—C14—H14C	109.5
C1—C2—Cl1	118.6 (2)	C19—C18—C17	112.4 (2)
O2—C1—O1	120.2 (3)	C19—C18—H18A	109.1
O2—C1—C2	131.1 (3)	C17—C18—H18A	109.1
O1—C1—C2	108.7 (3)	C19—C18—H18B	109.1
O3—C5—C6	107.9 (2)	C17—C18—H18B	109.1
O3—C5—C10	108.7 (2)	H18A—C18—H18B	107.9
C6—C5—C10	112.4 (2)	N1—C19—C18	110.9 (2)
O3—C5—H5	109.2	N1—C19—H19A	109.5
C6—C5—H5	109.2	C18—C19—H19A	109.5
C10—C5—H5	109.2	N1—C19—H19B	109.5
C5—C6—C7	109.6 (2)	C18—C19—H19B	109.5
C5—C6—C11	114.4 (2)	H19A—C19—H19B	108.0
C7—C6—C11	113.1 (2)	C15—C16—C17	111.3 (2)
C5—C6—H6	106.3	C15—C16—H16A	109.4
C7—C6—H6	106.3	C17—C16—H16A	109.4
C11—C6—H6	106.3	C15—C16—H16B	109.4
C8—C7—C6	112.4 (3)	C17—C16—H16B	109.4
C8—C7—H7A	109.1	H16A—C16—H16B	108.0
C6—C7—H7A	109.1	N1—C15—C16	111.1 (2)
C8—C7—H7B	109.1	N1—C15—H15A	109.4
C6—C7—H7B	109.1	C16—C15—H15A	109.4
H7A—C7—H7B	107.8	N1—C15—H15B	109.4
C5—C10—C9	112.4 (3)	C16—C15—H15B	109.4
C5—C10—H10A	109.1	H15A—C15—H15B	108.0
C9—C10—H10A	109.1	C18—C17—C20	112.2 (3)
C5—C10—H10B	109.1	C18—C17—C16	108.8 (2)
C9—C10—H10B	109.1	C20—C17—C16	111.1 (2)
H10A—C10—H10B	107.9	C18—C17—H17	108.2
C14—C11—C13	110.2 (4)	C20—C17—H17	108.2
C14—C11—C6	114.0 (3)	C16—C17—H17	108.2
C13—C11—C6	112.4 (3)	C17—C20—H20A	109.5
C14—C11—H11	106.5	C17—C20—H20B	109.5
C13—C11—H11	106.5	H20A—C20—H20B	109.5
C6—C11—H11	106.5	C17—C20—H20C	109.5
C8—C9—C10	108.7 (3)	H20A—C20—H20C	109.5
C8—C9—C12	112.9 (4)	H20B—C20—H20C	109.5
C10—C9—C12	110.2 (3)	C3—N1—C15	123.8 (2)
C8—C9—H9	108.3	C3—N1—C19	123.74 (19)
C10—C9—H9	108.3	C15—N1—C19	112.4 (2)
C12—C9—H9	108.3	C4—O3—C5	115.85 (16)
C7—C8—C9	111.0 (3)	C1—O1—C4	109.6 (2)
C7—C8—H8A	109.4	C9—C12—H12A	109.5
C9—C8—H8A	109.4	C9—C12—H12B	109.5
C7—C8—H8B	109.4	H12A—C12—H12B	109.5
C9—C8—H8B	109.4	C9—C12—H12C	109.5
H8A—C8—H8B	108.0	H12A—C12—H12C	109.5
C11—C13—H13A	109.5	H12B—C12—H12C	109.5

### Hydrogen-bond geometry (Å, °)

**Table:**

*D*—H···*A*	*D*—H	H···*A*	*D*···*A*	*D*—H···*A*
C4—H4···O2^i^	0.98	2.44	3.376 (3)	160
C18—H18B···O2^ii^	0.97	2.54	3.393 (4)	147

Symmetry codes: (i) −*x*+2, *y*+1/2, −*z*+1/2; (ii) *x*, *y*+1, *z*.
